# Wiskott-Aldrich Syndrome (WAS) and Dedicator of Cytokinesis 8- (DOCK8) Deficiency

**DOI:** 10.3389/fped.2019.00451

**Published:** 2019-11-05

**Authors:** Michael H. Albert, Alexandra F. Freeman

**Affiliations:** ^1^Dr. von Hauner University Children's Hospital, Ludwig-Maximilians Universität, Munich, Germany; ^2^National Institute of Allergy and Infectious Diseases, NIH, Bethesda, MD, United States

**Keywords:** HSCT, Wiskott-Aldrich syndrome (WAS), DOCK8 deficiency, eczema, infection

## Abstract

Both Wiskott-Aldrich syndrome (WAS) and dedicator of cytokinesis 8 (DOCK8) deficiency are primary immunodeficiency diseases caused by mutations in genes that result in defective organization of the cytoskeleton in hematopoietic tissues. They share some overlapping features such as a combined immunodeficiency, eczema and a predisposition to autoimmunity and malignancy, but also have some unique features that make them relatively easy to diagnose by clinical means. Both diseases can be cured by HSCT in a large proportion of patients. In WAS it is sometimes difficult to establish an indication for HSCT due to the large variability of disease severity, while HSCT is probably indicated in all patients affected by DOCK8 deficiency. There is considerably more published HSCT experience for WAS than for DOCK8 deficiency, but many open questions remain, which will be discussed in this review.

## Keypoints

- HSCT achieves excellent cure rates in WAS and DOCK8, but has known potential risks.
- For classic WAS patients, early HSCT is absolutely indicated. Due to the extreme variance of disease severity, determining the best treatment approach for milder affected WAS patients remains a challenge.
- HSCT is the treatment of choice for DOCK8 deficiency due to the early mortality and high incidence of significant infections and malignancy.
- Long term follow-up is needed to determine the risk of malignancy in DOCK8 deficiency post HSCT and the long-term outcome of vascular disease.

## Wiskott-Aldrich Syndrome

### Introduction

The Wiskott-Aldrich syndrome (WAS) occurs in males with hemizygous mutations in the X-chromosomal *WAS* gene. The fact that the WAS protein, which is absent or defective in WAS patients, is a critical regulator of the cytoskeleton and is expressed in all hematopoietic cell lineages, helps explain the multi-faceted manifestations of the disease. These include eczema, a combined immunodeficiency, thrombocytopenia, autoimmunity and a predisposition to mostly hematopoietic malignancy (1–4).

A striking feature of WAS is the extreme variability of disease severity. It ranges from infants with severe immunodeficiency, catastrophic bleeding complications and a severely reduced life expectancy to patients with no symptoms except thrombocytopenia and a presumably normal life expectancy (5, 6). Patients have been classified according to their disease severity as either classic WAS or X-linked thrombocytopenia, somewhat depending on the type of mutation, the presence of residual WAS protein, and a severity score. However, there is currently no reliable biomarker to predict disease severity. The WAS score is of limited usefulness for treatment decisions, also because the autoimmunity and malignancy can develop at any age including in otherwise mildly affected patients. This has implications for the recommended treatment modality for individual patients, as will be discussed below.

### Indication for HSCT

It is widely accepted that for patients with a classic WAS phenotype consisting of a clinically relevant immunodeficiency and thrombocytopenia with or without eczema, an allogeneic hematopoietic stem cell transplantation (HSCT) is absolutely indicated. This should be carried out as soon as the diagnosis is established, the best donor has been identified, and the patient's condition is optimized, which is typically not before 1 year of age. Development of autoimmune/autoinflammatory phenomena or malignancy also should be considered as a strong indication for HSCT.

For patients with a milder phenotype, the decision to proceed to HSCT is a much more difficult one, as some of those can have a normal life expectancy. Nevertheless, patients with an initial mild phenotype also have a high incidence of severe disease related complications—which presumably negatively affects their quality of life (5). For example, the sudden development of autoimmune kidney disease with consequent organ damage may make HSCT impossible or very risky. The incidences of autoimmunity or malignancy in “mild” patients is not negligible and has been estimated at about 30 and 25% at 40 years of age, respectively (5). Therefore, the HSCT indication in these milder patients should be re-evaluated on a regular basis and careful counseling should be performed taking into account factors such as family preference, patient age and ability to consent, donor availability, and fertility preservation.

### HSCT Approach

**Figure 1:**
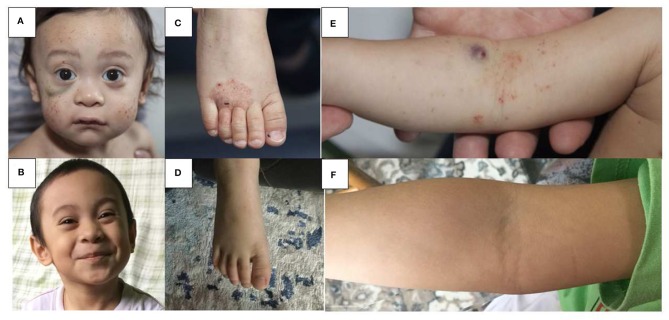
Skin findings pre and post HSCT in WAS. Multiple petechiae and hematoma in a 18 months old boy pre-HSCT **(A)** and 36 months post HLA-haploidentical HSCT **(B)**. Hemorrhagic eczema in the same boy pre-HSCT **(C,E)** and 36 months post HSCT **(D,F)**.

WAS was one of the first diseases treated by HSCT in 1968 (7) and since then many retrospective single and multi-center studies have analyzed HSCT outcome in WAS with generally encouraging results and complete reversal of the disease phenotype (Figure 1). However, some post HSCT complications such as autoimmune cytopenias (usually transient) have been reported to occur in up to 15% of patients after HSCT for WAS (8, 9). The most relevant studies reporting HSCT results for WAS are summarized in Table 1A.

**Table 1A:** Relevant published HSCT studies in WAS.

**References**	**Publication year**	**Type of study**	**Number of patients**	**Year of HSCT**	**Predominant conditioning regimen**	**OS**	**cGVHD**	**Other important findings**
Filipovich et al. (10)	2001	Retrospective, multi-center, registry-based	170	1968–1996	not reported	70% (5 years) MSD 87% MUD 71% MMFD 52%	13% limited 6% extensive	OS after MUD HSCT in patients <5 years equal to MSD HSCT.
Ozsahin et al. (9)	2008	Retrospective, multi-center, registry-based	96	1979–2001	Bu/Cy	This study included only patients who survived 2 years after HSCT. Of those, 97% survived long-term.	n/a	Post HSCT autoimmunity strongly associated with mixed chimerism.
Moratto et al. (8)	2011	Retrospective, multi-center	194	1980–2009	Bu/Cy	84% (5 years) after 2000: 90% (5 years)	6%	Mixed chimerism associated with autoimmunity.Myeloid chimerism correlates with post HSCT platelet counts.
Pai et al. (11)	2006	Retrospective, single center	23	1990–2005	Bu/Cy	78%	0%	Deaths only occurred in MMFD transplants or patients with a pre HSCT score of 5.
Balashov et al. (12)	2018	Retrospective, single center	32 (only MUD and MMFD)	2012–2017	Treo/Flu/Mel	91%	3% (limited only)	All patients transplanted with TCRa/b/CD19-depleted grafts.Recipient pre-conditioning with G-CSF and plerixafor reduced the incidence of poor platelet recovery or graft failure from 39% to 0%
Elfeky et al. (13)	2018	Retrospective, single center	34	1996–2016	Treo/Flu	100%	3% (limited only)	Myeloid chimerism correlates with post HSCT platelet counts.
Shin et al. (14)	2012	Retrospective, single center	47	1990–2009	Bu/Cy	81%	13% limited 9% extensive	HSCT from 2000 to 2009 and age <2 years associated with superior survival-
Stepensky et al. (15)	2013	Retrospective, multi-center	14	1996–2011	Bu/Cy (n = 6)	64%	14% (limited only)	Children conditioned with full dose Bu/Cy had a 100% EFS, compared with 25% for children conditioned with other regimens.

The seminal registry study of 170 WAS patients transplanted between 1968 and 1996 reported an overall survival (OS) of 87% with HLA-matched sibling donors (MSD), 71% with matched unrelated donors (MUD), and 52% with mismatched family donors (MMFD) (10). A large retrospective study conducted by the European Group of Blood and Marrow transplantation (EBMT) focused on long-term outcome of 96 patients who had survived at least 2 years post HSCT and were transplanted between 1979 and 2001 (9). Seven-year event-free survival (EFS) was 75%, when defining autoimmunity, active chronic GVHD (cGVHD), second HSCT, splenectomy after HSCT, death more than 2 years after HSCT, eczema lasting more than 2 years after transplant, severe infectious complications, and both disease- and transplant-related sequelae as events. EFS was superior with a MSD compared to a MUD or MMFD (88 vs. 71 vs. 55%). In univariate analysis only the presence of mixed chimerism, autoimmunity, cGVHD or splenectomy had a significant impact on EFS. This study also detected an increased frequency of post-HSCT autoimmunity in patients with incomplete donor chimerism, underlining the importance of complete donor engraftment in this disease. The study by Moratto et al. further highlighted the role of complete donor chimerism after HSCT in 186 patients transplanted between 1980 and 2009 mostly after busulfan/cyclophosphamide conditioning (11). In those transplanted from 2000 to 2009 5-year OS was 90%. Mixed chimerism in all lineages was associated with an increased risk of post-HSCT autoimmunity, and myeloid donor cell chimerism <50% was associated with persistent thrombocytopenia.

Several recent single-center reports have published excellent survival data, but larger, multi-center analyses reporting more recent results with state-of-the-art donor selection, conditioning regimens, and supportive care are currently missing (11–15). Thus, it can be postulated that the results of HSCT in WAS these days is even superior to what is reported in the available literature. A yet unpublished, retrospective study by the EBMT which analyzed 197 HSCTs for WAS with busulfan/fludarabine or treosulfan/fludarabine conditioning in a more recent era (2006–2016) found in an interim analysis excellent OS regardless of donor type (MSD, MUD, or MMFD). There was no difference of OS and EFS between the two conditioning regimens. Patients with treosulfan had a higher probability for mixed chimerism and required more secondary procedures (16).

### Alternative Approaches

Conservative treatment for classic WAS patients before HSCT should include bleeding prevention, antibiotic prophylaxis, immunoglobulin substitution, and eczema treatment. For those patients with a milder disease phenotype, all those conservative measures may also be indicated and can help to ameliorate disease burden. Platelet transfusions are necessary for life-threatening bleeding episodes and for surgical procedures. We discourage the routine prophylactic use of platelet transfusions in order to avoid development of anti-HLA or anti-platelet antibodies. Splenectomy is often an effective measure to elevate platelet counts and minimize the risk for bleeding, but it can increase the risk for fatal infectious complications and make a subsequent HSCT more risky (5). It should thus be reserved only for patients with a very mild phenotype who are fully vaccinated and expected to be compliant with life-long antibiotic prophylaxis to prevent post-splenectomy sepsis (17). The use of thrombopoietin agonists such as eltrombopag was found to result in moderate improvement of platelet counts and could be helpful to ameliorate the bleeding risk in selected patients (18). Autoinflammatory complications are usually responsive to steroid therapy, but other immunosuppressive agents may be used as well. Recently, anakinra was reported to be effective in two patients with severe autoinflammation (19, 20).

WAS is also one of the first diseases in which autologous stem cell gene therapy was clinically tested. Early optimism about good efficacy lessened when very high rates of insertional mutagenesis were observed with a retroviral vector (21). Consecutive studies with lentiviral vector constructs have demonstrated clinical improvement, immunological reconstitution, restoration of impaired platelet function, and no insertional mutagenesis (22–25). While bleeding tendency is greatly reduced after gene therapy, restoration of normal platelet numbers is generally not achieved (26). Because no alloreactivity can occur, this may become an alternative treatment option and marketing authorization is expected to be granted soon for lentiviral gene therapy. However, longer follow-up and comparative studies with HSCT will be needed to demonstrate long-term safety and persistent clinical benefit in the setting of a quasi-mixed chimeric state, which after HSCT was found to be associated with the development of autoimmunity and inferior outcome (see review on' Autologous stem cell-based gene therapy for inherited disorders: state-of-the-art and future prospects').

### Summary and Future Perspectives

Recent improvements of HSCT have led to excellent cure rates in WAS with HLA-matched as well as -haploidentical donors. HSCT at an early age is the treatment of choice for all classic WAS patients. It should also be discussed for patients with a milder phenotype, keeping in mind that it remains a sometimes dangerous procedure. This stresses the need for further improvement of HSCT and development of potentially safer therapies such as gene therapy. Given the portfolio of treatment options and the extreme variance of disease severity, it will be an ongoing challenge in the future to define the best modality for each and every patient and new biomarkers to predict disease outcome are urgently needed.

## DOCK8 Deficiency

### Introduction

Bi-allelic mutations in *DOCK8* were described in 2009 to cause a combined immunodeficiency previously referred to as autosomal recessive Hyper IgE syndrome (27, 28). Typical clinical features include eczema, allergies, recurrent oto-sinopulmonary infections, recurrent or severe viral skin infections, and malignancy (29). Clinical features often worsen with time resulting in end organ damage. For instance, recurrent pneumonias frequently lead to bronchiectasis, chronic HPV infection may lead to squamous cell carcinoma, poor EBV control may lead to lymphoma, and chronic cryptosporidium infection may lead to biliary sclerosis and cirrhosis (30). Affected individuals have a shortened life expectancy with about half dying before the age of 20 years, and about 80% having a life-threatening complication by age 20 years (29).

### Indication for HSCT

Due to the poor long-term prognosis of those with DOCK8 deficiency, HSCT is the treatment of choice. HSCT is curative and has been reported in about 100 individuals with overall good outcomes (31–34). Discussion about HSCT and donor evaluation should start soon after the diagnosis is made. Clinical manifestations of DOCK8 deficiency tend to worsen with age and chance of end organ toxicity increases, which will increase the risks of HSCT.

### HSCT Approach

**Figure 2:**
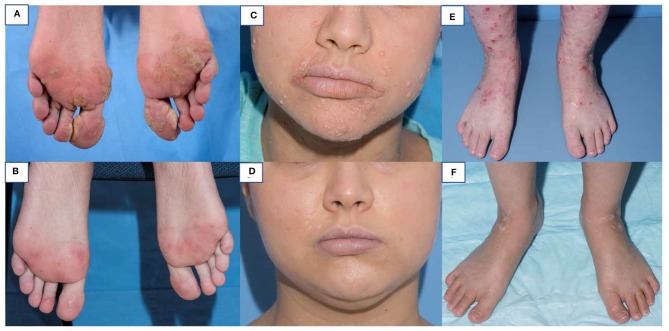
Skin findings pre and post HSCT in DOCK8 deficiency. Multiple warts on both feet of a 25 year old woman pre-HSCT **(A)** and 9 months post HSCT **(B)**. Extensive molluscum on the face of a 20 year old woman pre-HSCT **(C)** and 8 months post HSCT **(D)**. Severe eczema in a 13 year old girl pre-HSCT **(E)** and 6 months post-HSCT **(F)**.

In contrast to WAS, most available HSCT data in DOCK8 deficiency stem from one recent multi-center, international retrospective study of 81 transplanted patients. Overall survival was 84% at a median follow-up of 2 years (32). Patients ranged in age from 0.7 years to 27.2 years, and there were no clear survival differences based on age, with infection and infection related to GVHD being the most common causes of death. There was no survival difference between MRD and MUD transplants. The survival in patients receiving reduced toxicity conditioning with a treosulfan- or reduced-dose busulfan based regimen was overall better than in those with full busulfan-based myeloablation, and there was a significantly better outcome in transplants after 2010. Acute GVHD grade III-IV occurred in 11% of patients, and moderate to severe chronic GVHD in 6%. GVHD contributed to 5 of the 13 reported deaths. Chimerism at last follow-up was >90% in 88% of patients and graft failure only occurred in two patients, one after a cord blood transplant with non-myeloablative conditioning, and one after HSCT from a T-cell depleted mismatched family donor. Overall disease phenotype was reversed with resolution or significant improvement of eczema, skin viral infections and respiratory tract infections (Figure 2). Allergy was slower to improve in some reports, likely due to conditioning-resistant IgE-producing recipient plasma cells (35). Although expression of DOCK8 protein is largely limited to hematopoietic cells, some expression in non-hematopoietic tissues could play a role in delayed or partial correction after HSCT of some disease manifestations such as vascular disease. Some of the successful transplants were performed in patients with significant pre-existing underlying disease. For instance, one patient with a successful HSCT from a MSD and busulfan/fludarabine had a preceding liver transplant for chronic sclerosing cholangitis leading to liver failure (31). Another patient had chemo-refractory EBV driven lymphoma with full resolution after an ablative regimen with fludarabine and busulfan conditioning (33). Twelve patients had a malignant disease pre-transplant, but only one died due to progressive lymphoma after HSCT. Secondary malignancy post HSCT was seen in one patient with thyroid cancer after total body irradiation (32).

Haploidentical HSCT has been successful for patients in whom a matched donor is not available. Seven patients receiving T cell replete bone marrow transplants and post-transplant cyclophosphamide were reported (36). Acute GVHD did occur in 4/7, with a maximum of grade III in one, and chronic GVHD was not seen. Significant infectious disease complications were not seen; viral reactivations with CMV and EBV occurred, but responded to standard therapies. Neutrophil engraftment was seen at a median of 15 days, and chimerism was >90% donor by 30 days for all. Six of seven patients survived to a median follow-up of 4 years. One death was related to underlying lung disease and substance abuse. An additional report was of a patient with a haplo-identical transplant with post-transplant cyclophosphamide preceded by liver transplant 2 months earlier complicated by failure to engraft, multiple infections, and sinusoidal obstruction syndrome leading to death (37). Haploidentical HSCT with TCRab/CD19 depletion of the peripheral blood stem cell graft is increasingly being performed as well, and has been reported successful in a 7 year old using treosulfan, fludarabine, thiotepa and alemtuzumab for conditioning (38). Haploidentical HSCT is possibly preferable to the use of cord blood donors for those with DOCK8 deficiency due to the high viral burden associated with this disease and the lack of anti-viral immune memory in cord blood T-cells. Two reported cord blood transplants were unsuccessful (32, 34). HSCT results for DOCK8 deficiency are summarized in Table 1B.

**Table 1B:** Published HSCT studies in DOCK8 deficiency including multiple patients.

**References**	**Publication year**	**Type of study**	**Number of patients**	**Year of HSCT**	**Predominant conditioning regimen**	**OS**	**cGVHD**	**Other important findings**
Al-Herz et al. (31)	2016	Retrospective, two centers	11	Unknown	Bu/Cy Bu/Flu	91%	none	Some food allergies persist. Resolution of infections with mixed chimerism
Aydin et al. (32)	2019	Retrospective, multi-center	81	1995–2015	Treo/Flu Bu/Flu	89% MRD 81% MUD	10%	Improved outcomes with RIC
Cuellar-Rodriguez J et al. (33)	2015	Prospective, single center	6	2012–2013	Bu/Flu	100%	None	Well tolerated myeloablative regimen
Gatz et al. (39)	2011	Retrospective, Single center	2	Approx 2007–2009	Mel/Flu Radiotherapy	100%	None	
Kuskonmaz et al. (40)	2018	Retrospective, single center	3	Approx 2011–2013	Bu/Cy	100%	None	
Shah et al. (36)	2017	Prospective, single center	7	2013–2015	Haplo-identical with Bu/Flu post-tx Cy	86%	None	Successful haplo-identical transplant without significant complications
Uygun et al. (34)	2017	Retrospective, single center	4	2013–2015	Bu/Flu/ATG	100%	None	Cord blood transplant with rejection; Increased viral complications may have been from use of ATG

*Bu, busulfan; Cy, cyclophosphamide; Flu, fludarabine; Mel, melphalan; Treo, treosulfan*.

Pre-HSCT assessment of the extent of end-organ disease and infectious burden is especially important to minimize transplant associated morbidity and mortality. As these patients can have CNS vasculopathy and vasculitis, including cerebral artery stenosis, aneurysm and Moya-Moya, it is prudent to screen patients with brain MRA or CT angiogram, as finding CNS vascular disease would impact blood pressure and platelet management through HSCT (29). Aortic aneurysm and calcifications are seen as well and can be investigated with imaging. Bronchiectasis is common, reported in 44% of patients in one study (29), and knowing the colonizing infections (bacterial, mold, pneumocystis) will help in terms of optimizing treatment for lung infection pre-HSCT and antibiotic selection during neutropenia and bronchiectasis flares. Eczema therapy should be optimized as well, for example with consideration of wet wrap therapies. Infection with cryptosporidium spp. can cause chronic biliary liver disease, even in the absence of diarrhea, and is often difficult to diagnose. We find PCR techniques to be more sensitive to detect cryptosporidium. Therapy relies largely on immune reconstitution, but we have used nitazoxanide therapy during HSCT until immune reconstitution and removal of immunosuppressants. CMV and EBV viremia may be present chronically or intermittently pre-HSCT, and CMV should be suppressed pre-HSCT, and HSV and VZV infections treated.

### Alternative Approaches

Currently no gene therapy or other potentially curative approach for DOCK8 deficiency exists or is in pre-clinical development, so the mainstay of non-HSCT care is supportive. It relies on prophylactic antimicrobials and frequently immunoglobulin replacement. Trimethoprim/sulfamethoxazole (TMP/SMX) is a good choice for prophylaxis as *Pneumocystis jirovecii* pneumonia is a risk for these patients. Dosing of TMP/SMX, however, should be considered with daily or twice daily administration to also cover recurrent sinopulmonary bacterial infections. Azithromycin as additional bacterial coverage should be considered in the setting of bronchiectasis, once pulmonary non-tuberculous mycobacterial infection is excluded. Airway clearance techniques should also be instituted if bronchiectasis is present to minimize infections. Acyclovir or valacyclovir prophylaxis should be considered for patients with recurrent or chronic HSV or VZV infections. Typical therapies for warts and molluscum are often unsuccessful. Interferon (IFN) alpha has been used with some success as adjunctive therapy to treat warts, molluscum or resistant HSV infections (41–43), but adverse effects such as depression and cytopenias need to be considered. Treatment of cryptosporidium is very difficult without immune reconstitution, and so prevention is key by avoiding contaminated water supply. Clofazimine is currently being tested in a randomized trial in immunocompromised subjects (43). Low suspicion for malignancy is required in DOCK8 patients, with particular attention to HPV related gynecological cancers, skin cancers, and lymphomas.

### Summary and Future Perspectives

HSCT is considered curative for DOCK8 deficiency, and with transplant regimens and supportive care improving, HSCT should be discussed at the time of diagnosis, ideally prior to development of significant end organ damage. However, some questions over the long-term complications and prognosis remain. The vasculopathy of DOCK8 deficiency remains poorly understood, and long-term studies following HSCT are needed. The arterial aneurysm and stenosis occasionally seen are thought to be driven by inflammation or viral infection, and the hope is that HSCT will therefore at least stabilize disease. Skin microbiome studies in DOCK8 deficiency reveal a high burden of virus, largely HPV, even in areas without apparent disease (44). The poor control of HPV as well as potentially poor tumor surveillance leads to the malignancy, and theoretically should be improved by HSCT. However, long-term surveillance will need to demonstrate whether the microbiome does normalize and whether the higher risk of malignancy diminishes.

## Author Contributions

MA and AF wrote the manuscript and approved the final version.

### Conflict of Interest

The authors declare that the research was conducted in the absence of any commercial or financial relationships that could be construed as a potential conflict of interest.
